# Improving risk prediction accuracy for new soldiers in the U.S. Army by adding self-report survey data to administrative data

**DOI:** 10.1186/s12888-018-1656-4

**Published:** 2018-04-03

**Authors:** Samantha L. Bernecker, Anthony J. Rosellini, Matthew K. Nock, Wai Tat Chiu, Peter M. Gutierrez, Irving Hwang, Thomas E. Joiner, James A. Naifeh, Nancy A. Sampson, Alan M. Zaslavsky, Murray B. Stein, Robert J. Ursano, Ronald C. Kessler

**Affiliations:** 1 Department of Psychology, Harvard University, Cambridge, MA USA; 2 Department of Health Care Policy, Harvard Medical School, Boston, MA USA; 3 Department of Psychological and Brain Sciences, Center for Anxiety and Related Disorders, Boston University, Boston, MA USA; 4 Department of Psychiatry, University of Colorado School of Medicine, and Rocky Mountain Mental Illness Research, Education, and Clinical Center, Denver Veterans Affairs Medical Center, Denver, CO USA; 5 Department of Psychology, Florida State University, Tallahassee, FL USA; 6 Center for the Study of Traumatic Stress, Department of Psychiatry, Uniformed Services University School of Medicine, Bethesda, MD USA; 7 Departments of Psychiatry and Family Medicine and Public Health, University of California San Diego, La Jolla, CA USA

**Keywords:** Army, Military, Predictive modeling, Risk assessment, Violence, Sexual assault

## Abstract

**Background:**

High rates of mental disorders, suicidality, and interpersonal violence early in the military career have raised interest in implementing preventive interventions with high-risk new enlistees. The Army Study to Assess Risk and Resilience in Servicemembers (STARRS) developed risk-targeting systems for these outcomes based on machine learning methods using administrative data predictors. However, administrative data omit many risk factors, raising the question whether risk targeting could be improved by adding self-report survey data to prediction models. If so, the Army may gain from routinely administering surveys that assess additional risk factors.

**Methods:**

The STARRS New Soldier Survey was administered to 21,790 Regular Army soldiers who agreed to have survey data linked to administrative records. As reported previously, machine learning models using administrative data as predictors found that small proportions of high-risk soldiers accounted for high proportions of negative outcomes. Other machine learning models using self-report survey data as predictors were developed previously for three of these outcomes: major physical violence and sexual violence perpetration among men and sexual violence victimization among women. Here we examined the extent to which this survey information increases prediction accuracy, over models based solely on administrative data, for those three outcomes. We used discrete-time survival analysis to estimate a series of models predicting first occurrence, assessing how model fit improved and concentration of risk increased when adding the predicted risk score based on survey data to the predicted risk score based on administrative data.

**Results:**

The addition of survey data improved prediction significantly for all outcomes. In the most extreme case, the percentage of reported sexual violence victimization among the 5% of female soldiers with highest predicted risk increased from 17.5% using only administrative predictors to 29.4% adding survey predictors, a 67.9% proportional increase in prediction accuracy. Other proportional increases in concentration of risk ranged from 4.8% to 49.5% (median = 26.0%).

**Conclusions:**

Data from an ongoing New Soldier Survey could substantially improve accuracy of risk models compared to models based exclusively on administrative predictors. Depending upon the characteristics of interventions used, the increase in targeting accuracy from survey data might offset survey administration costs.

**Electronic supplementary material:**

The online version of this article (10.1186/s12888-018-1656-4) contains supplementary material, which is available to authorized users.

## Background

Concerns exist about high rates of interpersonal violence, mental disorders, and suicidality among U.S. Army soldiers [1–4]. Although intensive preventive interventions have been developed in the civilian population and shown to reduce risk of some of these outcomes, including those involving physical and sexual violence (e.g., [5, 6]), cost-effective implementation of these interventions would require that they be delivered only to soldiers judged to be high-risk. It has been shown that useful risk targeting systems can be developed for these outcomes based on administrative data available for all U.S. Army soldiers using machine learning methods, with the small proportions of soldiers predicted to be at high risk by these systems accounting for substantial proportions of subsequently observed instances of the outcomes [7–13]. However, many known risk factors for these outcomes are not assessed in Army administrative records, raising the possibility that risk targeting could be improved by expanding the predictor sets to include information from such additional data sources as self-report surveys [13] and social media postings [14].

Given that the risk of many negative outcomes, including involvement in physical and sexual violence [4, 15, 16], is especially high in the early years of Army service, a survey carried out at the beginning of service might be especially useful in providing information that would help increase the accuracy of risk-targeting beyond the accuracy achieved by exclusively using administrative data as predictors. A New Soldier Survey (NSS) of this sort was administered as part of the Army Study to Assess Risk and Resilience in Servicemembers (Army STARRS) [17]. As reported previously [13], prediction models derived from NSS data found that the small proportions of new soldiers judged to be at high risk based on NSS predictors accounted for relatively high proportions of attempted suicides, psychiatric hospitalizations, positive drug screens, and several types of violent crime perpetration and victimization. For example, the 10% of new male soldiers estimated in cross-validated models to have highest risk of major physical violence perpetration in the early years of service accounted for 45.8% of actual acts of major physical violence in the sample.

To date, no results have been reported about the extent to which information obtained in the NSS could be used to increase the accuracy of predictions based exclusively on administrative data. Such increases might be especially large early in the Army career, when administrative data are sparse, with the predictive power of NSS data decreasing relative to that of administrative predictors over time. The current report presents the results of the first attempt to add data from the NSS survey to previously-developed predicted risk scores based on administrative data. We focus on predicting physical and sexual violence perpetration among males and sexual violence victimization among females during the early years of Army service because these are the three outcomes for which separate risk models based on NSS and administrative data were previously developed.

## Methods

### Sample

The NSS was administered to representative samples of new U.S. Army soldiers beginning Basic Combat Training (BCT) at Fort Benning, GA, Fort Jackson, SC, and Fort Leonard Wood, MO between April 2011 and November 2012. Recruitment began by selecting weekly samples of 200–300 new soldiers at each BCT installation to attend an informed consent presentation within 48 h of reporting for duty. The presenter explained study purposes, confidentiality, and voluntary participation, then answered all attendee questions before seeking written informed consent to give a self-administered computerized questionnaire (SAQ) and neurocognitive tests and to link these data prospectively to the soldier’s administrative records. These study recruitment and consent procedures were approved by the Human Subjects Committees of all Army STARRS collaborating organizations. The 21,790 NSS respondents considered here represent all Regular Army soldiers who completed the SAQ and agreed to administrative data linkage (77.1% response rate). Data were doubly-weighted to adjust for differences in survey responses among the respondents who did versus did not agree to administrative record linkage and differences in administrative data profiles between the latter subsample and the population of all new soldiers. More details on NSS weighting are reported elsewhere [18]. The sample size decreased with duration both because of attrition and because of variation in time between survey and end of the follow-up period. The sample included 18,838 men (decreasing to 16,479 by 12 months, 15,306 by 24 months, and 3729 by 36 months) and 2952 women (decreasing to 2300 by 12 months, 2094 by 24 months, and 687 by 36 months).

### Measures

#### Outcomes

Outcome data were abstracted from Department of Defense criminal justice databases through December 2014 (25–44 follow-up months after NSS completion). Dependent variables were defined as first occurrences of each of the three outcomes for which predictive models had previously been developed from both administrative data and NSS data: major physical violence (i.e., murder-manslaughter, kidnapping, aggravated arson, aggravated violence, or robbery) perpetration by men, sexual violence perpetration by men, and sexual violence victimization of women, each coded according to the Bureau of Justice Statistics National Corrections Reporting Program classification system [19]. The perpetration outcomes were defined from records of “founded” offenses (i.e., where the Army found sufficient evidence to warrant full investigation). The victimization outcome was defined using *any* officially reported victimization regardless of evidence.

#### Predictors

As reported in previous publications, separate composite risk scores for each outcome were developed based on models from either the STARRS Historical Administrative Data System (HADS) [8, 9, 12] or the NSS [13]. The details of building the models that generated these scores are reported in the original papers and will not be repeated here other than to say that they involved the use of iterative machine learning methods [20] with internal cross-validation to predict the outcomes over a one-month risk horizon in a discrete-time person-month data array [21]. The HADS models were developed using all the nearly 1 million soldiers on active duty during the years 2004–2009 and were estimated for all years of service rather than only for the first few years of service, whereas the NSS models were developed using the NSS sample. We then applied the coefficients from these models to the data from the soldiers in the present samples to generate composite prediction scores. Thus, each person-month had a single score from each model representing the predicted log odds of the outcome occurring (note that this score changed each month for the HADS models, but remained the same within each person for the NSS models because the NSS was administered only once). Each score was then standardized by a mean of 0 and variance of 1 in the total sample. These composite prediction scores were used as the input in the current analysis. In other words, for each of the models reported here, there were two possible two independent variables (plus their transformations and interactions): the standardized log odds of the event occurring according to the HADS model and the standardized log odds of the event occurring according to the NSS model.

The potential predictors selected for inclusion in the iterative model-building process for the HADS and NSS models operationalized 8 classes of variables found in prior studies to predict the outcomes: socio-demographics (e.g., age, sex, race-ethnicity), mental disorders (self-reported Diagnostic and Statistical Manual of Mental Disorders, 4th edition [DSM-IV] disorders in the NSS and medically recorded International Classification of Diseases [ICD] disorders in the HADS models), suicidality/non-suicidal self-injury (self-reported in the NSS and medically recorded in the HADS models), exposure to stressors (assessed in detail in the NSS models with questions about childhood adversities, other lifetime traumatic stressors, and past-year stressful life events and difficulties; assessed in the HADS models with a small number of available markers of financial, legal, and marital problems, information about deployment and stressful career experiences, and military criminal justice records of prior experiences with crime perpetration and victimization), military career information (for new soldiers, Armed Forces Qualification Test [AFQT] scores; physical profile system [PULHES] scores used to indicate medical, physical, or psychiatric limitations; enlistment military occupational specialty classifications; and a series of indicators of enlistment waivers; and for the HADS models, increasing information over the follow-up period about promotions, demotions, deployments, and other career experiences), personality (only in the NSS models), and social networks (only in the NSS models). Results of performance-based neurocognitive tests administered in conjunction with the NSS were also included in the NSS models [22]. More detailed descriptions of the HADS and NSS predictors, the final form of each model (i.e., the variables that were ultimately selected for inclusion by the algorithms), and predictive performance are presented in the original reports [8, 9, 12, 13].

### Analysis methods

Analysis was carried out remotely by Harvard Medical School analysts on the secure University of Michigan Army STARRS Data Coordination Center server. Given that respondents differed in number of months of follow-up, we began by inspecting observed outcome distributions by calculating survival curves using the actuarial method [23] implemented in SAS PROC LIFETEST [24]. We projected morbid risk to 36 months even though some new soldiers were followed for as long as 44 months because the number followed beyond 36 months was too small for stable projection. Discrete-time survival analysis with person-month the unit of analysis and a logistic link function [21] was then used to estimate a series of nested prediction models for first occurrence of each outcome. Models were estimated using SAS PROC LOGISTIC [24].

The model-testing process involved two steps: first, determining the best model using the HADS risk score only, and then finding the optimal strategy for combining NSS data with the best model from the first step. Specifically, we began with a model including only the composite predicted risk score based on the HADS (expressed as a predicted log odds standardized to have a mean of 0 and a variance of 1), controlling (as in all subsequent models) for time in service; we then estimated models including a quadratic effect of HADS risk score, an interaction of the risk score with time, and their combination. In the second step, we tested the effect of adding the NSS composite predicted risk score to the best HADS model, followed by combinations of a quadratic NSS term, an interaction of NSS score with HADS score, and an interaction of NSS risk score with historical time. Importantly, whereas the values of the NSS composite risk score did not change with time in service because the NSS was administered only once, the values of the HADS composite risk score did change due to the addition of new administrative data each month. We tested the significance of interactions between the composite risk scores and time in service to evaluate the assumption that the HADS composite risk score might become more important over time and the NSS composite risk score less important. Design-based Wald χ^2^ tests based on the Taylor series method [25] were used to select the best-fitting model for each outcome. This method took into consideration the weighting and clustering of the NSS data in calculating significance tests.

Once the best-fitting model for each outcome was selected, we exponentiated the logistic regression coefficients and their design-based standard errors for that model to create odds-ratios (ORs) and 95% confidence intervals (95% CIs). We then divided the sample into 20 separate groups (ventiles), each representing 5% of respondents ranked in terms of their risk scores in the best-fitting models, and calculated concentration of risk for each ventile: the proportions of observed cases of the outcome in each ventile. If the models were strong predictors, we would expect high concentration of risk in the upper ventiles. Concentration of risk was calculated and compared not only for the best-fitting models but also for the HADS-only models to determine the improvement in prediction strength achieved by adding information from the NSS rather than relying exclusively on HADS risk scores. We also calculated concentration of risk for the NSS-only models for comparative purposes. Finally, we calculated positive predictive value: the proportion of soldiers in each ventile that had the outcome over the follow-up period. As with morbid risk, positive predictive value was projected to 36 months using the actuarial method to adjust for the fact that the follow-up period varied across soldiers.

## Results

### Outcome distributions

**Fig. 1:**
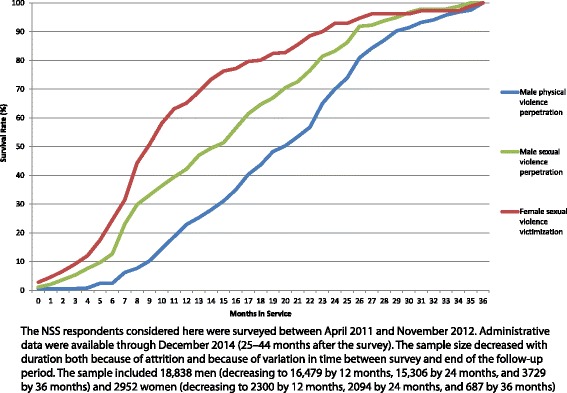
Survival curves for the outcomes over the 36-month follow-up period (*n* = 18,838 men and 2952 women)

A total of 186 male NSS respondents were accused of founded major physical violence perpetration and 132 of sexual violence perpetration by the end of the follow-up period, and 135 female NSS respondents reported sexual violence victimization over the same time period. These numbers correspond to incidence estimates per 1000 person-years of 4.5 for male physical violence perpetration, 3.1 for male sexual violence perpetration, and 19.5 for female sexual violence victimization. 36-month morbid risks per 1000 soldiers are 10.8 for male physical violence perpetration, 7.7 for male sexual violence perpetration, and 42.6 for female sexual violence victimization (computed using the actuarial method [23] implemented in SAS PROC LIFETEST [24]). Survival curves show that all outcomes were much less likely to occur in the first months of service, when new soldiers are in training, than later in the follow-up period (Fig. 1). Median (interquartile range) months-to-occurrence were 20 (13–25) for male physical violence perpetration, 14 (7–22) for male sexual violence perpetration, and 9 (6–15) for female sexual violence victimization.

### Correlations between predictions based on the separate NSS and HADS models

**Fig. 2:**
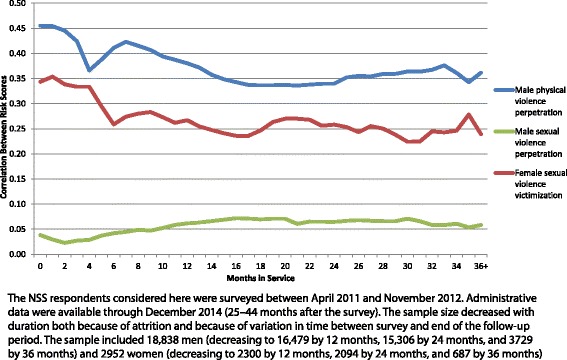
Pearson correlations between composite risk scores based on the HADS and the NSS by month in the NSS sample (*n* = 18,838 men and 2952 women)

The correlations between HADS and NSS composite risk scores varied over time because of monthly changes in the administrative variables used to generate the HADS predictions. Median (interquartile range) within-month Pearson correlations between the two scores were .36 (.34–.38) for major physical violence perpetration among men, .06 (.05–.07) for sexual violence perpetration among men, and .26 (.24–.27) for sexual violence victimization among women (Table 1). The magnitudes of the associations between the two composite risk scores decreased over time for physical violence perpetration and sexual violence victimization, with Pearson correlations of −.78 and − .84 between number of months in service and magnitude of the within-month association between the two scores. The associations increased over time, in comparison, for sexual violence perpetration, *r* = .84 (Fig. 2).

**Table 1:** Pearson correlations between composite risk scores based on the HADS and the NSS by month in the NSS sample (*n* = 18,838 men and 2952 women)^a^

	Physical violence perpetration^b^	Sexual violence perpetration^b^	Sexual violence victimization^c^
Month	r	r	r
0	0.46	0.04	0.34
1	0.45	0.03	0.35
2	0.44	0.02	0.34
3	0.42	0.03	0.33
4	0.37	0.03	0.33
5	0.39	0.04	0.30
6	0.41	0.04	0.26
7	0.42	0.04	0.27
8	0.42	0.05	0.28
9	0.41	0.05	0.28
10	0.39	0.05	0.27
11	0.39	0.06	0.26
12	0.38	0.06	0.27
13	0.37	0.06	0.26
14	0.36	0.07	0.25
15	0.35	0.07	0.24
16	0.34	0.07	0.24
17	0.34	0.07	0.24
18	0.34	0.07	0.25
19	0.34	0.07	0.26
20	0.34	0.07	0.27
21	0.34	0.06	0.27
22	0.34	0.06	0.27
23	0.34	0.06	0.26
24	0.34	0.06	0.26
25	0.35	0.07	0.25
26	0.36	0.07	0.24
27	0.35	0.07	0.26
28	0.36	0.07	0.25
29	0.36	0.07	0.24
30	0.36	0.07	0.22
31	0.36	0.07	0.22
32	0.37	0.06	0.24
33	0.38	0.06	0.24
34	0.36	0.06	0.25
35	0.34	0.05	0.28
36+	0.36	0.06	0.24
25% Quartile	0.34	0.05	0.24
Median	0.36	0.06	0.26
75% Quartile	0.39	0.07	0.27

^a^The NSS respondents considered here were surveyed between April 2011 and November 2012. Administrative data were available through December 2014 (25-44 months after the survey). The sample size decreased with duration both because of attrition and because of variation in time between survey and end of the follow-up period. The sample included 18,838 men (decreasing to 16,479 by 12 months, 15,306 by 24 months, and 3,729 by 36 months) and 2,952 women (decreasing to 2,300 by 12 months, 2,094 by 24 months, and 687 by 36 months)

^b^Males only

^c^Females only

### Relative fit of the base models and extensions

In the first analytic step, none of the expansions of the base HADS models for nonlinearities or interactions improved model fit in predicting either physical violence perpetration among men or sexual violence victimization among women. However, the addition of the NSS risk score improved model fit in both cases. We consequently focused on the additive model (i.e., HADS plus NSS composite risk score) for these outcomes. For sexual violence perpetration among men, however, model fit was improved by inclusion of the interaction between the NSS composite risk score and time since survey administration (χ^2^_2_ = 6.8, *p* = .034) relative to the additive model (Table 2). (See Additional file 1: Table S1, for odds ratios and chi-square values for all models tested.)

**Table 2:** Model fit statistics and model comparison tests (*n* = 18,838 men and 2952 women)^a,b^

		Male physical violence perpetration			Male sexual violence perpetration			Female sexual violence victimization		
		df	χ^2^	*p*	df	χ^2^	*p*	df	χ^2^	*p*
I. Models^c^										
M1	T	32	93.4	<.0001	34	47.7	0.059	29	498.1	<.0001
M2	T + A	33	638.4	<.0001	35	152.8	<.0001	30	521.7	<.0001
M3	T + A + T*A	35	711.0	<.0001	37	185.5	<.0001	32	851.3	<.0001
M4	T + A + A^2^	34	497.2	<.0001	36	99.5	<.0001	31	549.7	<.0001
M5	T + A + T*A + A^2^	36	524.9	<.0001	38	138.4	<.0001	33	866.1	<.0001
M6	Best model for A (Ba) + S	34	578.5	<.0001	36	268.9	<.0001	31	473.4	<.0001
M7	Ba + S + T*S	36	597.2	<.0001	38	308.8	<.0001	33	574.7	<.0001
M8	Ba + S + S^2^	35	576.5	<.0001	37	253.3	<.0001	32	635.5	<.0001
M9	Ba + S + T*S + S^2^	37	604.7	<.0001	39	268.9	<.0001	34	678.9	<.0001
M10	Ba + S + A*S	–	–	–	37	238.1	<.0001	–	–	–
M11	Ba + S + A*S + T*S	–	–	–	39	291.5	<.0001	–	–	–
II. Model Differences										
M2-M1	A	1	259.2	<.0001	1	42.8	0.000	1	15.3	0.000
M3-M2	T*A	2	1.5	0.469	2	2.2	0.339	2	0.6	0.744
M5-M4	T*A	1	3.0	0.085	1	2.9	0.089	1	0.2	0.690
M4-M2	A^2^	2	2.1	0.351	2	2.3	0.324	2	0.3	0.848
M5-M3	A^2^	1	3.9	0.050	1	3.2	0.073	1	0.0	0.959
M6-Ba	S	1	24.2	0.000	1	54.1	0.000	1	43.3	<.0001
M7-M6	T*S	2	0.5	0.797	2	6.8	0.034	2	0.3	0.871
M9-M8	T*S	1	0.4	0.543	1	0.4	0.530	1	0.2	0.629
M8-M6	S^2^	2	0.5	0.796	2	6.0	0.050	2	0.3	0.877
M9-M7	S^2^	1	0.4	0.527	1	1.3	0.253	1	0.3	0.616
M11-M10	T*S	–	–	–	2	5.9	0.053	–	–	–
M11-M7	A*S	–	–	–	1	3.6	0.059	–	–	–

*Abbreviations*: *Time (T)* time since survey administration (main effects of T dummy coded with each month), *S* predicted log odds from New Soldier Survey (NSS), *A* predicted log odds from Historical Administrative Data System (HADS), *A*^*2*^ the square of A, T*A the interaction between T and A (where T is dummy coded with indicator variables for 13–24 months and 25+ months), *Ba* predictors from best model among models 1 through 5, T*S interaction between T and S (T dummy coded with indicator variables for 13–24 months and 25+ months), *S*^*2*^ S-squared, S*A interaction of S and A

^a^The NSS respondents considered here were surveyed between April 2011 and November 2012. Administrative data were available through December 2014 (25-44 months after the survey). The sample size decreased with duration both because of attrition and because of variation in time between survey and end of the follow-up period. The sample included 18,838 men (decreasing to 16,479 by 12 months, 15,306 by 24 months, and 3,729 by 36 months) and 2,952 women (decreasing to 2,300 by 12 months, 2,094 by 24 months, and 687 by 36 months).

^b^Although the same sample of soldiers was used for both male outcomes, the number of person-months differed because we predicted first occurrence of each outcome, and each soldier was censored after the month when the outcome first occurred, termination of service, or December 2014, whichever came first. Number of person-months was 543,603 for male physical assault perpetration, 543,636 for male sexual assault perpetration, and 75,772 for female sexual assault victimization.

^c^Out of M1-M5, M2 was the best model for each outcome; M6-M11 add NSS predicted log odds to the best model (Ba) from HADS data alone. The final best models were M6 for physical violence perpetration and sexual violence victimization and M7 for sexual violence perpetration

### Coefficients in the best-fitting models

Inspection of the odds ratios (ORs) of univariate models with either the NSS or HADS composite risk scores as the only predictors shows that each score is associated with significantly increased odds of each outcome, with ORs relatively comparable in magnitude for NSS (OR = 1.9–2.1) and HADS (OR = 1.5–2.5). Due to their collinearity, individual predictors’ ORs are lower but remain significant in the two additive models that include both composite risk scores as predictors (HADS OR = 2.1 for physical violence perpetration and 1.3 for sexual violence victimization; NSS OR = 1.6 for physical violence perpetration and 1.8 for sexual violence victimization). In the model for sexual violence perpetration, the HADS composite risk score is significant (OR = 1.4) and stable over the follow-up period, whereas the NSS composite risk score is a significant predictor in the first 12 months of service (OR = 2.3), decreases but remains significant during the second year of service (months 13–24; OR = 1.7), and becomes nonsignificant beyond the second year of service (months 25+; OR = 1.3) (Table 3).

**Table 3:** Odds ratios for univariate and best-fitting models (*n* = 18,838 men and 2,952 women)^a,b^

	Male physical violence perpetration		Male sexual violence perpetration		Female sexual violence victimization	
	OR	(95% CI)	OR	(95% CI)	OR	(95% CI)
I. NSS univariate model	2.1	(1.8–2.5)	1.9	(1.6–2.3)	1.9	(1.6–2.1)
II. HADS univariate model	2.5	(2.2–2.8)	1.5	(1.3–1.7)	1.7	(1.3–2.2)
III. Best-fitting model						
NSS	1.6	(1.3–1.9)	–	–	1.8	(1.5–2.1)
HADS	2.1	(1.9–2.5)	1.4	(1.2–1.6)	1.3	(1.0–1.8)
NSS*Time (0–12 Mo)	–	–	2.3	(1.8–2.9)	–	–
NSS*Time (13–24 Mo)	–	–	1.7	(1.3–2.1)	–	–
NSS*Time (25+ Mo)	–	–	1.3	(0.8–2.2)	–	–

*Abbreviations*: *OR* odds ratio, *CI* confidence interval, *NSS* standardized predicted log odds from model based on survey data, *HADS* standardized predicted log odds from model based on administrative data

^a^The NSS respondents considered here were surveyed between April 2011 and November 2012. Administrative data were available through December 2014 (25-44 months after the survey). The sample size decreased with duration both because of attrition and because of variation in time between survey and end of the follow-up period. The sample included 18,838 men (decreasing to 16,479 by 12 months, 15,306 by 24 months, and 3,729 by 36 months) and 2,952 women (decreasing to 2,300 by 12 months, 2,094 by 24 months, and 687 by 36 months).

^b^All coefficients were estimated controlling for time (number of months in service)

### Concentration of risk and positive predictive value in the best-fitting models

**Fig. 3:**
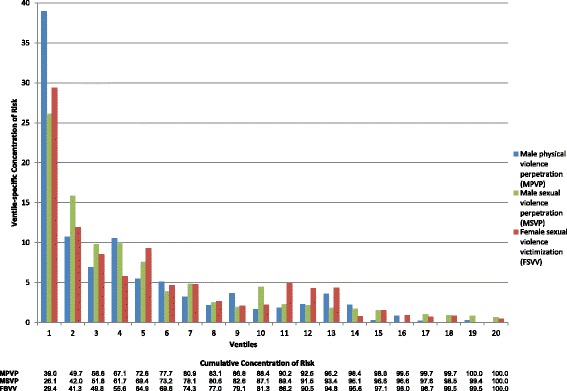
Concentration of risk by ventiles for best model of each outcome

Concentration of risk was strongly elevated compared to the 5% of observed cases expected by chance in the top 3 predicted risk ventiles of all three best-fitting models (Fig. 3). 39.5% of observed physical violence perpetration, 26.1% of sexual violence perpetration, and 29.4% of sexual violence victimization occurred among the 5% of soldiers in the top risk ventiles for those outcomes (Table 4). Between 49.8% (sexual violence victimization) and 56.3% (physical violence perpetration) of observed cases of the outcomes occurred among the 15% of soldiers in the top three risk ventiles.

**Table 4:** Performance of univariate and best-fitting models (*n* = 18,838 men and 2952 women)^a^

	Top ventile (5%)				Top two ventiles (10%)				Top three ventiles (15%)			
	HADS-only	NSS-only	Best^b^	Proportional Improvement Best/HADS^c^	HADS-only	NSS-only	Best^b^	Proportional Improvement Best/HADS^c^	HADS-only	NSS-only	Best^b^	Proportional Improvement Best/HADS^c^
I. Concentration of Risk (%)												
Male physical violence perpetration	33.9	24.8	39.5	16.6	45.2	38.4	50.2	11.2	52.3	48.7	56.3	7.8
Male sexual violence perpetration	20.7	21.8	26.1	26.0	32.4	33.8	42.0	29.6	35.5	46.5	51.8	45.9
Female sexual violence victimization	17.5	27.6	29.4	67.9	32.1	38.3	41.3	28.7	47.6	47.9	49.8	4.8
II. Observed Positive Predictive Value^d^												
Male physical violence perpetration	2.9	2.2	3.4	17.2	2.0	1.7	2.2	10.0	1.5	1.4	1.6	6.7
Male sexual violence perpetration	1.2	1.2	1.5	25.0	0.9	1.0	1.2	33.3	0.7	0.9	1.0	42.9
Female sexual violence victimization	6.8	10.6	11.5	69.1	6.3	7.4	8.1	28.6	6.2	6.2	6.5	4.8
III. Projected Positive Predictive Value^e^												
Male physical violence perpetration	68.2	50.4	79.1	16.0	46.0	39.2	51.0	10.9	35.5	33.1	38.4	8.2
Male sexual violence perpetration	27.6	29.0	34.6	25.4	21.6	22.6	27.9	29.2	15.8	20.7	23.0	45.6
Female sexual violence victimization	151.6	225.9	241.7	59.4	139.8	163.8	176.4	26.2	138.3	139.0	144.6	4.6

*Abbreviations*: *NSS-only* prediction from model based on survey data alone, *HADS-only* prediction from model based on administrative data alone

^a^The NSS respondents considered here were surveyed between April 2011 and November 2012. Administrative data were available through December 2014 (25–44 months after the survey). The sample size decreased with duration both because of attrition and because of variation in time between survey and end of the follow-up period. The sample included 18,838 men (decreasing to 16,479 by 12 months, 15,306 by 24 months, and 3729 by 36 months) and 2952 women (decreasing to 2300 by 12 months, 2094 by 24 months, and 687 by 36 months)

^b^Additive model for physical violence perpetration and sexual violence victimization; model including interaction with time for sexual violence perpetration

^c^Proportional increase in concentration of risk or positive predictive value of the best model relative to the HADS-only model

^d^Observed cases per 1000 person-months

^e^Number of cases per 1000 soldiers projected to 36 months

These proportions were for the most part meaningfully higher than those achieved by using only the HADS predicted risk score (Table 4). For example, the 39.5% concentration of risk of physical violence perpetration among soldiers in the top risk ventile of the best-fitting model was proportionally 16.6% greater than the 33.9% concentration of risk among soldiers in the top risk ventile of the model based only on the HADS predicted risk score (i.e., 39.5/33.9). Three of these 9 proportional improvements (i.e., the top 3 ventiles for each of 3 outcomes) were less than 15% (4.8–11.2%). Four others were 15–30% (16.6–29.6%). The largest two were 45.9% and 67.9%.

Despite the high concentrations of risk in the top predicted risk ventiles, positive predictive value was low even in the highest risk ventiles due to the rarity of the outcomes. In any given month, 3.4/1000 male soldiers in the highest predicted risk ventile of physical violence perpetration were accused of that outcome, 1.5/1000 male soldiers in the highest predicted risk ventile of sexual violence perpetration were accused of that outcome, and 11.5/1000 female soldiers in the highest predicted risk ventile of sexual violence victimization experienced that outcome. However, cumulative positive predictive value projected over the first 36 months of service was considerably higher, between 34.6 and 241.7/1000 soldiers in the highest risk ventile across the outcomes.

## Discussion

Prediction of all three outcomes considered here was improved, in some cases substantially so, by adding information from the NSS predicted risk score to information from the HADS predicted risk score. One would expect this improvement to shrink somewhat in out-of-sample performance due to the fact that the NSS predicted risk score was developed in the same sample as it was applied. However, a counter-balancing consideration is that incremental prediction accuracy might increase beyond the level found here if an NSS survey became a routine part of Army accession, as the sample available for analysis would then be large enough for disaggregated analyses of individual predictors from both administrative and survey data rather than requiring the use of the composite predicted risk scores we were forced to use here because of the small NSS sample size.

We found unexpectedly that the strength of the NSS predicted risk scores remained stable over the time period of the study for physical violence perpetration and sexual violence victimization. This suggests that the NSS tapped into relatively stable individual differences in risk factors for these two outcomes rather than situational risk factors that became less relevant over time. A review of the most important predictors making up the NSS predicted risk scores showed, consistent with this interpretation, that these variables are dominated by measures of personality, history of pre-enlistment lifetime psychopathology, and history of pre-enlistment lifetime trauma exposures, most notably prior sexual violence victimization among women and prior involvement in violence among men [13]. For sexual violence perpetration, however, the NSS risk score was no longer a significant predictor after the end of the second year in service (i.e., in months 25 and beyond). This could simply be a function of greater uncertainty in the model as time progresses (as the confidence interval for the odds ratio at months 25+ still contains relatively large values), or it could reflect a true decrease in the predictive strength over time. Regardless, the NSS data were valuable for predicting the majority of sexual violence perpetration outcomes, because 83.2% of reported assaults occurred within the first two years of service.

It is less clear why the HADS predicted risk scores did not increase in strength over time, as administrative information about soldiers became richer over time. One plausible interpretation is that the early months of service, when administrative records are sparse, are also characterized by lower prevalence of the outcomes considered here, as new soldiers are more closely supervised during Basic Combat Training (BCT; the first 10–16 weeks in service) and Advanced Individual Training (2–12 months after completion of BCT) so that opportunities for violence are lower. Administrative records become richer after the end of training, at which time prevalence of violence outcomes also increases. The extent to which the temporal consistencies in prediction accuracy continue beyond the early years of service studied here is unclear, although it seems unlikely that the prediction accuracy of survey reports obtained from 18-year-old new soldiers will maintain constant strength over many years in service. This question will be the focus of ongoing analyses of the STARRS data as the NSS cohort ages.

Even though the addition of NSS data improved prediction of all outcomes beyond the HADS predictions, the question remains whether the magnitudes of these improvements are large enough to justify implementing an ongoing NSS for all new soldiers. The answer depends on the number of interventions the Army might want to implement (which could involve many more than the three outcomes considered in this report), the proportional increases in concentration of risk of a composite risk score using the NSS as well as the HADS compared to the HADS alone at the thresholds selected by the Army for intervention implementation, and the costs, benefits, and competing risks of those interventions in relation to the costs of implementing an ongoing NSS. Uncertainties about these values make it impossible to calculate these cost-benefit ratios here, but these are the calculations the Army needs to make if it is interested in using evidence-based standards for targeting preventive interventions for high-risk new soldiers. If so, future research might also consider other data sources that could be added beyond an ongoing NSS to improve prediction accuracy even further over the accuracy of models based exclusively on administrative predictors.

The performance of these models is on par with, or better than, other attempts to use machine learning or more traditional methods to predict risk of crime (e.g., [26, 27]), but the accuracy is nonetheless intermediate in strength. Consequently, using these predictions as a basis for decision-making (whether with or without NSS predictor) requires that the benefits of the action taken for those accurately classified as high-risk outweigh the costs to those misclassified as high-risk. For instance, it would certainly be beneficial to deliver a reasonably low-cost intervention that does no harm to those to whom it is administered, but has some effect on reducing interpersonal violence, to a group of soldiers identified as high risk using these models. However, classification might not be accurate enough to deliver an intervention with high per-person expense, or one that causes some kind of harm (e.g., stigma, limiting career advancement) to those who are misclassified.

In addition to their implications for informing U.S. Army decision-making regarding data collection and use, these findings may be relevant to other researchers using machine learning methods to predict various outcomes for individual humans. In this study, even an extremely rich passively-collected administrative data set was no substitute for querying individuals directly about psychologically relevant variables. Administrative/institutional data are often abundant and incur relatively low additional cost to collect, so they are have formed the typical feature sets used in machine learning algorithms. However, prediction may be considerably improved through the addition of self-report data, especially (1) when an outcome is partly psychologically driven, and consequently, subjective information may be a powerful predictor, and/or (2) when important predictors in administrative data sets may be noisy or inaccurate because they are not fully captured by institutional systems (e.g., health events when no medical care was sought, covert antisocial behaviors). The control conferred when administering self-report questionnaires is an additional advantage; researchers can select the variables they consider to be most essential, and questionnaires can be designed in such a way that data require little processing prior to use in algorithms.

## Conclusions

Self-report information can substantially improve prediction of risk for interpersonal violence beyond the information available in administrative databases for Regular Army soldiers early in their careers. The U. S. Army may benefit from ongoing administration of self-administered questionnaires to new soldiers. Other researchers may want to consider collecting self-report data to augment administrative/institutional data sets when developing machine learning algorithms, especially to predict psychologically driven outcomes.

## Additional file

Additional file 1: **Table S1**. Odds ratios and chi-square values for all models (*n* = 18,838 men and 2952 women). Results of all models tested and indices of fit. (DOCX 23 kb)

## Abbreviations

AFQT: Armed Forces Qualification Test
BCT: Basic Combat Training
DSM-IV: Diagnostic and Statistical Manual of Mental Disorders, 4th edition
HADS: Historical Administrative Data System
ICD: International Classification of Diseases
NSS: New Soldier Survey
OR: Odds ratio
PUHLES: Physical profile system
SAQ: Self-administered questionnaire
STARRS: Study to Assess Risk and Resilience in Service members
