# Supplementary material for: Improving risk prediction accuracy for new soldiers in the U.S. Army by adding self-report survey data to administrative data
Source: BMC Psychiatry. 2018 Apr 3;18:87. doi: 10.1186/s12888-018-1656-4 (PMC5883887; doi:10.1186/s12888-018-1656-4)
Supplement: Supplementary file 1 — Table S1. Odds ratios and chi-square values for all models (n = 18,838 men and 2952 women). Results of all models tested and indices of fit. (DOCX 23 kb) [file 12888_2018_1656_MOESM1_ESM.docx]

| **Table S1. Odds ratios and chi-square values for all models (n = 18,838 men and 2,952 women)^1,2^** | | | | | | | | | | | | | | |
| --- | --- | --- | --- | --- | --- | --- | --- | --- | --- | --- | --- | --- | --- | --- |
|  |  |  |  |  |  |  |  |  |  |  |  |  |  |  |
|  | **Male physical violence**  **perpetration** | | | |  | **Male sexual violence**  **perpetration** | | | |  | **Female sexual violence victimization** | | | |
|  | **OR** | **(95% CI)** | **χ^2^** | ***p*** |  | **OR** | **(95% CI)** | **χ^2^** | ***p*** |  | **OR** | **(95% CI)** | **χ^2^** | ***p*** |
| Model 1: S | | | | |  |  |  |  |  |  |  |  |  |  |
| S | 2.1 | (1.8-2.5) | 90.1 | 0.000 |  | 1.9 | (1.6-2.3) | 61.0 | 0.000 |  | 1.9 | (1.6-2.1) | 90.9 | 0.000 |
| Model 2: A |  |  |  |  |  |  |  |  |  |  |  |  |  |  |
| A | 2.5 | (2.2-2.8) | 259.2 | 0.000 |  | 1.5 | (1.3-1.7) | 42.8 | 0.000 |  | 1.7 | (1.3-2.2) | 15.3 | 0.000 |
| Model 3: A + Time (T)*A |  |  |  |  |  |  |  |  |  |  |  |  |  |  |
| A | 2.1 | (1.6-2.8) | 31.7 | 0.000 |  | 1.5 | (1.2-2.0) | 10.8 | 0.001 |  | 1.8 | (1.3-2.4) | 14.0 | 0.000 |
| A*13-24 months | 1.2 | (0.8-1.7) | 0.8 | 0.367 |  | 0.9 | (0.7-1.2) | 0.5 | 0.473 |  | 0.9 | (0.5-1.5) | 0.2 | 0.661 |
| A*25+ months | 1.2 | (0.9-1.6) | 1.5 | 0.218 |  | 1.1 | (0.8-1.6) | 0.4 | 0.548 |  | 0.8 | (0.4-1.5) | 0.5 | 0.470 |
| Model 4: A + A^2^ | | |  |  |  |  |  |  |  |  |  |  |  |  |
| A | 2.9 | (2.3-3.7) | 80.5 | 0.000 |  | 2.0 | (1.3-3.0) | 10.9 | 0.001 |  | 1.6 | (1.2-2.3) | 8.2 | 0.004 |
| A^2^ | 1.0 | (0.9-1.0) | 3.0 | 0.085 |  | 0.9 | (0.8-1.0) | 2.9 | 0.089 |  | 1.0 | (0.9-1.1) | 0.2 | 0.690 |
| Model 5: A + T*A + A^2^ | | | |  |  |  |  |  |  |  |  |  |  |  |
| A | 2.4 | (1.7-3.5) | 25.0 | 0.000 |  | 2.1 | (1.3-3.5) | 9.5 | 0.002 |  | 1.8 | (1.0-3.0) | 4.6 | 0.032 |
| A*13-24 months | 1.2 | (0.8-1.9) | 1.2 | 0.281 |  | 0.9 | (0.6-1.3) | 0.6 | 0.440 |  | 0.9 | (0.5-1.7) | 0.1 | 0.709 |
| A*25+ months | 1.3 | (0.9-1.9) | 2.1 | 0.148 |  | 1.2 | (0.7-2.0) | 0.5 | 0.490 |  | 0.8 | (0.4-1.7) | 0.3 | 0.571 |
| A^2^ | 0.9 | (0.9-1.0) | 3.9 | 0.050 |  | 0.9 | (0.8-1.0) | 3.2 | 0.073 |  | 1.0 | (0.9-1.1) | 0.0 | 0.959 |
| Model 6: A + S |  |  |  |  |  |  |  |  |  |  |  |  |  |  |
| A | 2.1 | (1.9-2.5) | 122.7 | 0.000 |  | 1.4 | (1.2-1.6) | 30.0 | 0.000 |  | 1.3 | (1.0-1.8) | 4.1 | 0.044 |
| S | 1.6 | (1.3-1.9) | 24.2 | 0.000 |  | 1.9 | (1.6-2.2) | 54.1 | 0.000 |  | 1.8 | (1.5-2.1) | 43.3 | 0.000 |
| Model 7: A + S + T*S |  |  |  |  |  |  |  |  |  |  |  |  |  |  |
| A | 2.1 | (1.9-2.5) | 123.4 | 0.000 |  | 1.4 | (1.2-1.6) | 30.3 | 0.000 |  | 1.3 | (1.0-1.8) | 4.1 | 0.043 |
| S | 1.7 | (1.2-2.3) | 10.1 | 0.001 |  | 2.3 | (1.8-2.9) | 51.7 | 0.000 |  | 1.8 | (1.5-2.2) | 39.8 | 0.000 |
| S*13-24 months | 0.9 | (0.6-1.3) | 0.4 | 0.534 |  | 0.7 | (0.5-1.0) | 3.6 | 0.059 |  | 0.9 | (0.6-1.5) | 0.1 | 0.802 |
| S*25+ months | 1.0 | (0.7-1.4) | 0.0 | 0.834 |  | 0.6 | (0.3-1.0) | 4.6 | 0.032 |  | 0.8 | (0.4-1.6) | 0.3 | 0.611 |
| Model 8: A + S + S^2^ |  |  |  |  |  |  |  |  |  |  |  |  |  |  |
| A | 2.1 | (1.9-2.5) | 122.8 | 0.000 |  | 1.4 | (1.2-1.6) | 30.3 | 0.000 |  | 1.3 | (1.0-1.8) | 4.1 | 0.043 |
| S | 1.6 | (1.3-2.1) | 16.5 | 0.000 |  | 2.0 | (1.5-2.7) | 20.4 | 0.000 |  | 1.8 | (1.5-2.3) | 26.9 | 0.000 |
| S^2^ | 1.0 | (0.9-1.1) | 0.4 | 0.543 |  | 1.0 | (0.9-1.1) | 0.4 | 0.530 |  | 1.0 | (0.9-1.1) | 0.2 | 0.629 |
| Model 9: A + S + T*S + S^2^ | | | |  |  |  |  |  |  |  |  |  |  |  |
| A | 2.1 | (1.9-2.5) | 123.6 | 0.000 |  | 1.4 | (1.3-1.6) | 30.8 | 0.000 |  | 1.3 | (1.0-1.8) | 4.1 | 0.042 |
| S | 1.8 | (1.2-2.5) | 10.3 | 0.001 |  | 2.7 | (1.7-4.2) | 20.4 | 0.000 |  | 1.9 | (1.4-2.6) | 18.0 | 0.000 |
| S^2^ | 1.0 | (0.9-1.1) | 0.4 | 0.527 |  | 0.9 | (0.8-1.0) | 1.3 | 0.253 |  | 1.0 | (0.9-1.1) | 0.3 | 0.616 |
| S*13-24 months | 0.9 | (0.6-1.3) | 0.4 | 0.534 |  | 0.7 | (0.4-1.1) | 3.0 | 0.084 |  | 0.9 | (0.6-1.6) | 0.1 | 0.801 |
| S*25+ months | 1.0 | (0.6-1.4) | 0.0 | 0.845 |  | 0.5 | (0.3-0.9) | 4.8 | 0.028 |  | 0.8 | (0.4-1.7) | 0.2 | 0.620 |
| Model 10: A + S + A*S | | | |  |  |  |  |  |  |  |  |  |  |  |
| A | 2.3 | (1.9-2.8) | 76.4 | 0.000 |  | 1.5 | (1.3-1.8) | 33.2 | 0.000 |  | 1.5 | (1.1-2.0) | 8.5 | 0.004 |
| S | 1.7 | (1.4-2.1) | 23.8 | 0.000 |  | 2.0 | (1.6-2.3) | 54.1 | 0.000 |  | 1.9 | (1.6-2.4) | 43.2 | 0.000 |
| A*S | 0.9 | (0.8-1.0) | 1.9 | 0.173 |  | 0.9 | (0.8-1.0) | 5.5 | 0.019 |  | 0.9 | (0.8-1.0) | 2.3 | 0.131 |
| Model 11: A + S + A*S + T*S | | | |  |  |  |  |  |  |  |  |  |  |  |
| A | 2.3 | (1.9-2.8) | 77.6 | 0.000 |  | 1.5 | (1.3-1.7) | 34.3 | 0.000 |  | 1.5 | (1.2-2.0) | 10.3 | 0.001 |
| S | 1.8 | (1.2-2.5) | 10.3 | 0.001 |  | 2.4 | (1.9-3.0) | 51.1 | 0.000 |  | 2.1 | (1.7-2.6) | 55.6 | 0.000 |
| A*S | 0.9 | (0.8-1.0) | 1.9 | 0.163 |  | 0.9 | (0.9-1.0) | 3.6 | 0.059 |  | 0.9 | (0.7-1.0) | 4.3 | 0.039 |
| S*13-24 months | 0.9 | (0.6-1.3) | 0.3 | 0.596 |  | 0.7 | (0.5-1.0) | 3.0 | 0.084 |  | 0.9 | (0.5-1.4) | 0.3 | 0.584 |
| S*25+ months | 1.0 | (0.7-1.5) | 0.0 | 0.964 |  | 0.6 | (0.4-1.0) | 4.1 | 0.043 |  | 0.7 | (0.3-1.6) | 0.7 | 0.394 |
|  |  |  |  |  |  |  |  |  |  |  |  |  |  |  |

Abbreviations: Time (T), time since survey administration (the main effects of T were dummy coded with each month but treated as a continuous variable coded in the range 0-36 in estimating the interactions of composite predicted risk scores with time); S, predicted log odds from New Soldier Survey (NSS); A, predicted log odds from Historical Administrative Data System (HADS); A^2^, the square of A; T*A, the interaction between T and A (where T is dummy coded with indicator variables for 13-24 months and 25+ months); T*S, interaction between T and S (where T is dummy coded with indicator variables for 13-24 months and 25+ months); S^2^, S-squared; S*A, interaction of S and A.

^1^The NSS respondents considered here were surveyed between April 2011 and November 2012. Administrative data were available through December 2014 (25-44 months after the survey). The sample size decreased with duration both because of attrition and because of variation in time between survey and end of the follow-up period. The sample included 18,838 men (decreasing to 16,479 by 12 months, 15,306 by 24 months, and 3,729 by 36 months) and 2,952 women (decreasing to 2,300 by 12 months, 2,094 by 24 months, and 687 by 36 months).

^2^All coefficients were estimated controlling for time (number of months in service).
